# Intermittent Theta Burst Stimulation Combined with Cognitive Training in Mild Cognitive Impairment: Protocol for an 18-Week Randomised, Double-Blind, Sham-Controlled Feasibility Pilot Study (The iCog.X Boost Trial)

**DOI:** 10.3390/mps9050130

**Published:** 2026-09-01

**Authors:** Rose Mery Bou Merhy, Rocco Cavaleri, Ghufran Alhassani, Vincent Oxenham, Najwa-Joelle Metri, Daniel Hochstrasser, Mark I. Hohenberg, Kujan Nagaratnam, Kawaljit Singh, Genevieve Z. Steiner-Lim

**Affiliations:** 1 NICM Health Research Institute, Western Sydney University, Penrith, NSW 2751, Australia; 22137273@student.westernsydney.edu.au (R.M.B.M.); r.cavaleri@westernsydney.edu.au (R.C.); j.metri@westernsydney.edu.au (N.-J.M.); d.hochstrasser@westernsydney.edu.au (D.H.); 2 Brain Stimulation and Rehabilitation (BrainStAR Lab), School of Health Sciences, Western Sydney University, Penrith, NSW 2751, Australia; ghufran.alhassani@westernsydney.edu.au; 3 Translational Health Research Institute (THRI), Western Sydney University, Penrith, NSW 2751, Australia; 4 School of Psychological Sciences, Lifespan Health and Wellbeing Research Centre, Macquarie University, Sydney, NSW 2109, Australia; vincent.oxenham@mq.edu.au; 5 Department of Neurology, Royal North Shore Hospital, Sydney, NSW 2065, Australia; 6 School of Medicine, Western Sydney University, Penrith, NSW 2751, Australia; m.hohenberg@westernsydney.edu.au; 7 School of Medicine, The University of Notre Dame, Sydney, NSW 2007, Australia; kujeswaran.nagaratnam@nd.edu.au; 8 Aged Care Specialist Medical Service, Campbelltown, NSW 2560, Australia; kawaljit@acsmedicalservice.com.au; 9 Department of Psychiatry and Psychotherapy, Jena University Hospital, 07743 Jena, Germany; 10 German Center for Mental Health (DZPG), Partner Site Jena, 07743 Jena, Germany

**Keywords:** cognitive training, mild cognitive impairment (MCI), transcranial magnetic stimulation (TMS), randomised controlled trial (RCT), dementia prevention

## Abstract

**Introduction:** Mild cognitive impairment (MCI) is a transitional stage between normal ageing and dementia, affecting ~35% of adults aged 70+ and carrying a 15–18% annual risk of progression. Cognitive training (CT) shows promise but outcomes are variable and transfer to daily function is unclear; intermittent theta burst stimulation (iTBS) may enhance prefrontal neuroplasticity. Few studies have evaluated whether combining CT and iTBS in MCI is practical, acceptable, and safe. **Methods:** This 18-week, randomised, double-blind, sham-controlled feasibility pilot trial will recruit 44 individuals with MCI, randomised 1:1 to CT + active iTBS or CT + sham iTBS. Participants complete 14 CT sessions over 10 weeks; accelerated iTBS is delivered in five sessions per day over five consecutive days at 75% resting motor threshold to the left DLPFC using neuronavigation, with reversed-coil sham. Primary outcomes are feasibility, acceptability, adherence, and safety; secondary (exploratory) outcomes are cognitive, functional, and psychological; tertiary (mechanistic) outcomes are EEG- and TMS-derived neurophysiological measures. The trial is not powered for efficacy: feasibility and neurophysiological outcomes will be analysed descriptively, while secondary outcomes will be analysed using linear mixed-effects models. **Ethics and dissemination:** Ethical approval has been granted by the Western Sydney University HREC (H16500); written informed consent will be obtained before enrolment. **Trial registration number:** ACTRN12625000952448.

## 1. Strengths and Limitations of This Study

- This feasibility trial evaluates the combined delivery of intermittent theta burst stimulation and cognitive training in individuals with mild cognitive impairment.
- The primary focus on feasibility, acceptability, adherence, and safety outcomes directly addresses the gap in the evidence base prior to undertaking a definitive efficacy trial.
- Neurophysiological outcomes using EEG and TMS provide exploratory mechanistic data to inform the design of a future fully powered trial.
- The mixed delivery format, including home-based cognitive training, enhances accessibility and real-world feasibility for older adults.
- As a feasibility pilot, this study is not powered to detect treatment efficacy; cognitive and neurophysiological findings are hypothesis-generating only and should be interpreted with caution.

## 2. Introduction

Mild cognitive impairment (MCI), formally known as Mild Neurocognitive Disorder according to DSM-5-TR criteria [1], is characterised by a measurable decline in cognitive abilities, particularly in memory and executive functions, without significantly impacting daily functioning. MCI affects around 35% of adults over 70 [2], and carries a 15–18% annual risk of progression to dementia, which increases longitudinally [3]. MCI thus represents a critical window for intervention, with the potential to delay or prevent further deterioration and preserve independence.

Current pharmacological treatments, such as cholinesterase inhibitors and memantine, have demonstrated limited efficacy and are not approved for MCI due to modest benefit and side effects [4]. Anti-amyloid agents like aducanumab and lecanemab have shown minimal clinical efficacy and are restricted to amyloid-positive individuals [5]. These drugs carry risks including cerebral oedema and haemorrhage [6]. Non-pharmacological strategies therefore remain the primary mode of intervention.

Non-pharmacological approaches targeting modifiable risk factors such as lifestyle and cognition have received increasing attention [7], though cognitive benefits of interventions such as physical activity remain inconsistent [8]. Consequently, more targeted approaches that directly enhance cognition and support daily functioning are needed.

Cognitive training (CT) has emerged as a prominent intervention for MCI. CT involves structured tasks aimed at improving specific cognitive domains through repeated practice [9]. Strategy-based CT focuses on compensatory techniques such as visual imagery to support daily functioning [10], while computerised CT delivers adaptive exercises targeting memory, attention, and processing speed [11,12]. Combining both forms may offer complementary benefits in instrumental activities of daily living (iADLs) by addressing compensatory and restorative mechanisms in an engaging and more personalised approach [13].

While numerous studies show that CT can improve cognitive performance in memory, attention, and executive function [14], outcomes remain variable. Neuroimaging has demonstrated increased activation in frontoparietal regions and enhanced neural connectivity post-training, supporting underlying neuroplastic changes [15]. However, CT adherence can be challenging in MCI due to task repetition and fatigue [16], and the transfer of gains to real-world functional improvements remains inconsistent [17]. This highlights the need to optimise CT to better translate cognitive gains into daily life benefits.

Neuromodulatory techniques such as repetitive transcranial magnetic stimulation (rTMS) may enhance CT by priming cortical regions associated with cognitive control [18]. rTMS is a non-invasive brain stimulation that modulates cortical excitability by delivering electromagnetic pulses to the brain [19]. When applied to the dorsolateral prefrontal cortex (DLPFC), high-frequency rTMS can facilitate long-term potentiation-like effects and strengthen cognitive functions such as working memory [20]. Despite these benefits, standard rTMS protocols are time-consuming (up to 60 min per session for several weeks), which can limit feasibility and increase dropout, especially in older adults. Studies have highlighted that reducing session time may improve adherence and clinical outcomes [21].

Intermittent theta burst stimulation (iTBS) offers a more feasible alternative. iTBS mimics the brain’s natural theta rhythms (5 Hz) associated with memory and learning by delivering brief, high-frequency bursts in under 3 min [22]. This protocol enhances synaptic plasticity and cortical excitability [23], and when applied to the left DLPFC, has been shown to produce preliminary signals of cognitive benefit in individuals with AD and MCI [24]. Additionally, iTBS is safe and well-tolerated [25], making it a potentially feasible therapeutic strategy for people with MCI.

Neurophysiological measures provide complementary indices of the neural changes that may accompany combined iTBS and CT. iTBS applied to the left DLPFC is hypothesised to induce long-term potentiation-like plasticity that increases cortical excitability and modulates intracortical inhibition. TMS paired-pulse paradigms (short- and long-interval intracortical inhibition, SICI and LICI) provide measures of these intracortical inhibitory processes. EEG provides complementary markers of network function during rest and cognitive processing, including event-related potential components (e.g., N2 and P3) that are commonly associated with inhibitory control and attentional allocation during the Go/NoGo task, as well as aperiodic (1/*f*) activity derived from resting EEG, as a proposed marker of cortical excitation/inhibition balance. Examining these measures alongside cognitive outcomes may provide preliminary insight into whether intervention-related cognitive change is accompanied by changes in cortical excitability, inhibition, and neurocognitive processing. Given the feasibility design, these neurophysiological analyses are exploratory and hypothesis-generating rather than definitive tests of mechanism.

Preliminary studies combining CT with rTMS have reported cognitive benefits compared with CT alone [26,27]. However, most trials used lengthy, standard rTMS protocols rather than accelerated iTBS protocols. Few have specifically explored iTBS paired with CT in MCI populations. Existing studies are limited by small sample sizes, unstructured training protocols, no formal assessment of feasibility, acceptability, and safety, and the absence of neurophysiological outcome measures [28]. This feasibility pilot therefore aims to determine whether a combined CT+iTBS intervention can be delivered as planned in an MCI population and whether predefined progression criteria are met, while generating preliminary estimates of cognitive and neurophysiological outcomes to inform a future definitive trial.

## 3. Aims and Hypotheses

The primary aim is to evaluate the feasibility, acceptability, adherence, and safety of combining CT with iTBS in people with MCI. Secondary (exploratory) aims are to examine signals of change in cognition, functional capacity, psychological wellbeing, and health-related quality of life. Tertiary (mechanistic) aims are to explore neurophysiological changes associated with active versus sham iTBS using EEG and TMS, including measures of cortical excitability, inhibition, and cognitive processing. As an exploratory feasibility pilot, the trial is not powered to test efficacy. Secondary and tertiary outcomes are hypothesis-generating and will inform outcome selection and sample-size estimation for a future definitive trial. We hypothesise that the trial will meet the predefined progression criteria in Table 1 (recruitment, eligibility, adherence, tolerability, acceptability, and retention) and that the intervention will be delivered safely.

## 4. Methods

### 4.1. Study Design and Setting

This is an 18-week, randomised, double-blind, sham-controlled, parallel-group feasibility pilot trial conducted across two Western Sydney University research sites: NICM Health Research Institute and the School of Health Sciences. The trial comprises two arms: CT + active iTBS and CT + sham iTBS with 1:1 randomisation. Participants will undergo in-person assessments and stimulation sessions during the initial intervention phase, followed by a home-based cognitive training program using a secure, university-hosted online platform developed using the Flask web framework (Python 3.11). Data will be stored securely via REDCap (Vanderbilt University, Nashville, TN, USA), with remote coaching delivered through Zoom Video Communications, Inc. (San Jose, CA, USA) to support adherence. This protocol was developed in accordance with the SPIRIT guidelines and adheres to the CONSORT extension for randomised pilot and feasibility trials, as well as the principles of ICH E6 Good Clinical Practice.

### 4.2. Participants

Adults aged 55 to 85 years meeting the study criteria for MCI will be recruited. Eligibility criteria were developed in accordance with international TMS safety guidelines in research settings [29] and are summarised in Table 2.

### 4.3. Interventions

#### 4.3.1. iTBS Stimulation Protocol

Participants allocated to the active stimulation group will receive iTBS over the left DLPFC across 5 consecutive weekdays. Each day comprises 5 iTBS sessions, spaced 10 min apart, totalling 25 sessions and 3000 pulses/day. Stimulation will be delivered using a MagPro x100 (MagVenture A/S, Farum, Denmark) at NICM Health Research Institute, or a MagStim Super Rapid2 Plus1 (MagStim Co Ltd., Whitland, UK) at the School of Health Sciences, each with a 70 mm figure-of-eight coil. Coil placement is guided by the Brainsight Neuronavigation System (Rogue Research Inc., Montreal, QC, Canada), using the BeamF3 algorithm to localise F3 coordinates according to the international 10–20 EEG system. The coil is held tangentially at 45° to induce a posterior–anterior current.

Resting motor threshold (RMT) will be determined using single-pulse TMS over the primary motor cortex. Surface EMG will be recorded from the right first dorsal interosseous (FDI) muscle using Ag/AgCl electrodes (active electrode on the muscle belly, reference electrode on the right olecranon), amplified ×32,000, bandpass-filtered (20–1000 Hz), and digitised at 2000 Hz via a Micro1401 Data Acquisition System and Signal software (Cambridge Electronic Design, Cambridge, UK). The motor hotspot will be defined as the scalp site where at least 5 out of 10 pulses elicit a motor-evoked potential (MEP) ≥ 50 µV in the relaxed FDI [31]. RMT will be reassessed daily, with stimulation intensity set at 75% of RMT.

Each iTBS session follows the standard protocol: bursts of 3 pulses at 50 Hz repeated at 5 Hz (every 200 ms), delivered in 2 s trains with 8 s inter-train intervals, for a total of 600 pulses per session lasting approximately 3 min. This protocol adheres to international safety and reporting guidelines for TMS [29] and the TMS Methodological Checklist by Chipchase et al. [32]. Participants may continue stable medications that do not affect cortical excitability, but substances known to lower seizure threshold (antipsychotics and recreational drugs) will be prohibited during the trial.

#### 4.3.2. Sham iTBS Intervention

Sham stimulation will use the same figure-of-eight coil as the active condition, rotated 180° so only the edge contacts the scalp, limiting magnetic field penetration while preserving some auditory and somatosensory sensations. This method has been validated as a sham condition in prior research [33]. Setup will be performed by the unblinded laboratory technician.

#### 4.3.3. Cognitive Training Intervention

The iCog.X Boost Program is a 10-week, structured CT intervention for individuals with MCI. It includes 14 training sessions, delivered across two phases: 5 in-person sessions followed by 9 online sessions delivered via a secure online platform. Participants also attend 2 small-group psychoeducation sessions via Zoom Video Communications, Inc. (San Jose, CA, USA), covering cognitive ageing, strategy use, and real-world cognitive management.

The program targets memory, attention, reasoning, and problem-solving, commonly affected in MCI [34]. It combines explicit strategies, progressive task difficulty, and real-life applications. Sessions include guided exercises, and real-world generalisation tasks to support daily implementation.

The in-person phase introduces core strategies before participants transition to the independent online phase, where sessions reinforce and consolidate these strategies through repeated practice. Task difficulty is dynamically adjusted based on accuracy, speed, and adherence to maintain optimal engagement without cognitive overload. Real-time coaching is provided.

To facilitate adherence, participants receive weekly email reminders and follow-up calls. After 2 consecutive missed sessions, additional contact attempts will be made via 2 calls and 1 email. Participants missing 3 consecutive sessions without responses are withdrawn. A 2-week grace period will be provided to allow catch-up.

**Figure 1:**
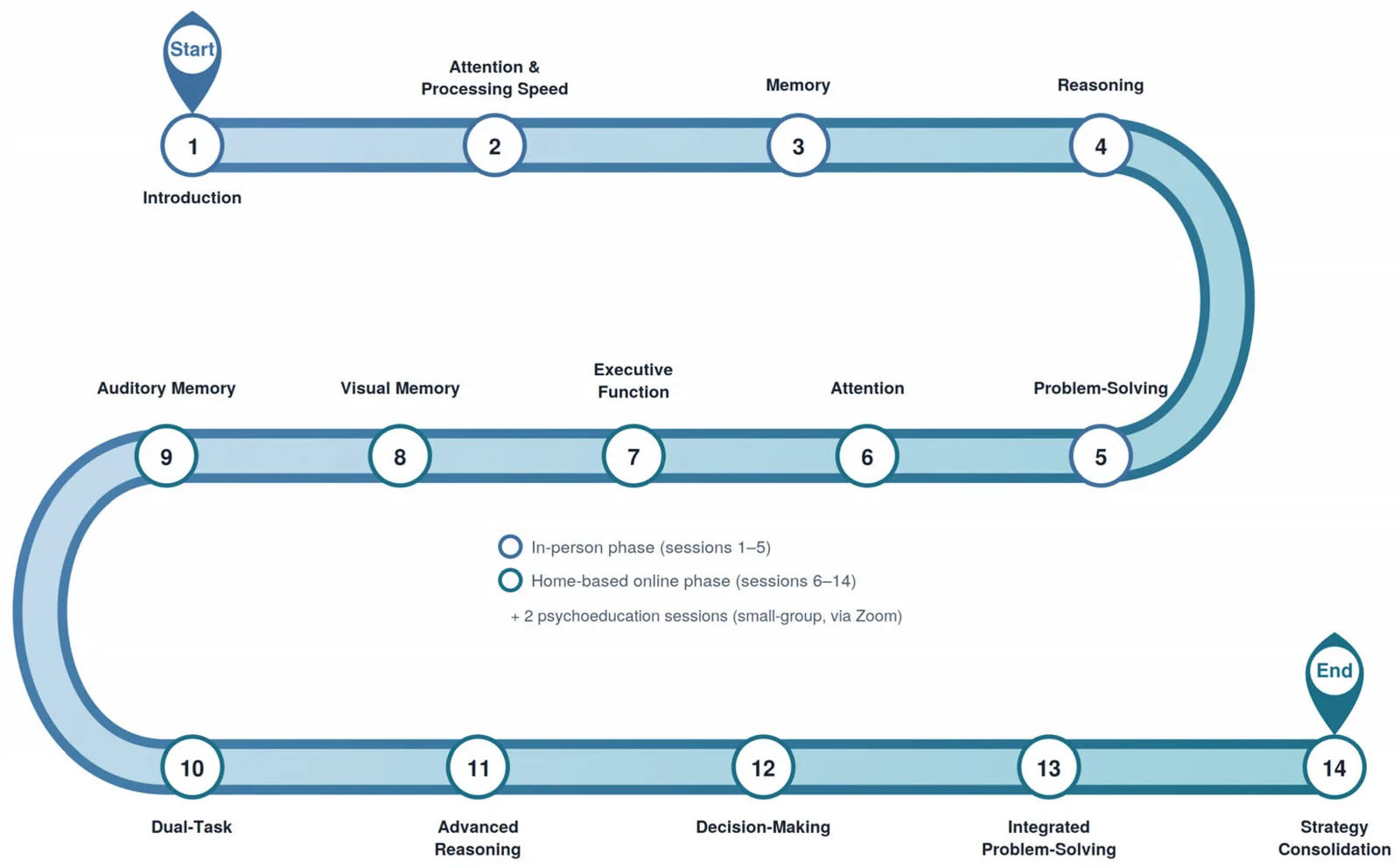
Overview of the cognitive training progression across the 14 sessions of the iCog.X Boost Program.

The intervention follows a scaffolded approach, gradually transitioning from foundational cognitive skills to higher-order executive functions. Following each session, participants complete home exercises to integrate strategies into daily life (e.g., writing and recalling a grocery list). The CT structure is illustrated in Figure 1 and a session-by-session breakdown is provided in Table 3.

Psychoeducation sessions cover cognitive changes in ageing, everyday challenges, and strategy application. Group delivery encourages experience sharing and social support, which may improve adherence, real-life transfer, and reduce isolation linked to cognitive decline in MCI [35].

### 4.4. Outcome Measures

Outcomes will be assessed at 5 timepoints: baseline (Week 0), post-iTBS (Day 5, Week 1), mid-intervention (Week 5), post-intervention (Week 10), and follow-up (Week 18). As this is a feasibility pilot trial, the primary outcomes are feasibility, acceptability, adherence, and safety. The predefined progression (“stop/go”) criteria in Table 1, spanning recruitment, eligibility, adherence, tolerability, acceptability, and retention, will determine whether to proceed to a full-powered definitive trial, while safety is monitored throughout. Each primary domain is operationalised below.

**Feasibility (recruitment and retention).** Recruitment feasibility will be reported descriptively, including the number of participants registering interest, the proportion screened, eligibility yield, and the time taken to reach the target sample; these indicators establish whether recruitment is viable within the study’s eligibility constraints ahead of a definitive trial. Retention will be expressed as the proportion of randomised participants completing the post-iTBS (Week 1), post-intervention (Week 10), and follow-up (Week 18) assessments, and it will be evaluated against the attrition thresholds in Table 1.

**Adherence.** Adherence will be quantified as the proportion of scheduled iTBS and CT sessions completed, against the predefined thresholds in Table 1. iTBS attendance will be verified against site logs, while home-based CT completion will be captured automatically through the online training platform on a per-session basis. Missed sessions and the reasons for non-completion will be documented, and patterns of adherence across the intervention period, including session completion over time, drop-off, and whether adherence differs between the initial in-person sessions and the subsequent home-based period, will be summarised descriptively.

**Acceptability.** Acceptability will be evaluated using a post-intervention satisfaction scale and semi-structured exit interviews examining perceived burden, comfort, and willingness to participate again, and will be judged against the acceptability threshold in Table 1.

**Safety and tolerability.** Safety will be monitored at every iTBS session and throughout the follow-up period; participants will be asked to report adverse sensations (e.g., headache, scalp discomfort, tingling, and fatigue), and all adverse events (AEs) will be reviewed for severity and relatedness and managed in accordance with the safety procedures described in Section 4.10. Tolerability will be operationalised as the proportion of withdrawals that are not attributable to intervention-related AEs, and will be evaluated against the threshold in Table 1.

Secondary outcomes include cognitive measures of verbal memory (CVLT-II), and episodic memory (RBANS Story Recall), executive function and processing speed (D-KEFS TMT; WAIS-IV Coding, Similarities; Digit Span), functional independence (HBA-FAQ), psychological well-being (PHQ-9, GAD-7, PSQI), and health-related quality of life (EQ-5D-5L). All measures were selected based on clinical relevance and psychometric properties (Supplementary Table S1). These outcomes are exploratory and are included not to establish efficacy but to estimate the magnitude and variability of potential change and to inform outcome selection and sample-size estimation for a future definitive trial. Exploratory signals of change will be characterised using effect size estimates (Cohen’s d) with means, standard deviations, and 95% confidence intervals.

Tertiary outcomes are mechanistic and include neurophysiological measures of cortical excitability and inhibition using paired-pulse TMS paradigms, including short- and long-interval intracortical inhibition (SICI, LICI), resting-state EEG and event-related potentials (ERPs) recorded during cognitive tasks. Given the feasibility design, these neurophysiological measures are exploratory and hypothesis-generating. They are intended to determine which markers can be feasibly acquired and are worth retaining as mechanistic outcomes in a definitive trial.

### 4.5. Neurophysiological Recording

Participants will undergo all assessments in a quiet, temperature-controlled room at either site. EEG data will be acquired using the Compumedics Okti^®^ 32-channel portable acquisition system (Compumedics Ltd., Abbotsford, VIC, Australia). Participants will be fitted with the Okti^®^ EEG recording apparatus and electrooculogram (EOG) electrodes and seated approximately 80 cm from a 21-inch monitor. Electrode placement follows the international 10–20 system across 32 channels (Fp1 through Oz, including AFz, FCz, M1 and M2). Signals will be referenced online to Cz, sampled at 1000 Hz, with a DC–70 Hz bandwidth and a 50 Hz notch filter. EOG electrodes will be placed vertically above/below the left eye, and horizontally 1 cm lateral to the outer canthi of each eye.

A brief EOG calibration task will precede EEG recording to facilitate later artefact correction using the Revised Aligned Artefact Average (RAAA) method [36]. Resting-state EEG will be recorded in two continuous 3 min blocks (eyes open/close), following validated protocols for ageing populations [37,38]. Spectral analysis will extract aperiodic (1/f) and periodic components as exploratory measures related to cortical excitability and inhibition [39,40].

Continuous EEG will also be recorded during an auditory Go/NoGo task designed to probe attention and cognitive control [41,42]. The task consists of two blocks of 150 pseudorandomised trials (70% Go, 30% NoGo), using 1000 Hz and 2000 Hz tones (80 ms, 15 ms rise/fall), with a 1250 ms stimulus onset asynchrony. Participants respond as quickly and accurately as possible to Go tones and withhold responses to NoGo tones. EEG data will be pre- and post-processed offline to extract ERPs using the HEADBOX Lab pipeline as published previously [43,44,45]. Temporal principal components analysis will be applied to derive N2 and P3 ERP component amplitudes, markers of inhibitory control and attentional allocation known to be sensitive to cognitive changes in MCI.

TMS assessments will include the determination of RMT. SICI and LICI will be assessed using standardised paired-pulse paradigms to probe intracortical inhibitory processes. The test stimulus will be calibrated to elicit an MEP of approximately 1 mV in amplitude in the FDI muscle of the right hand. For SICI, a 90% RMT conditioning stimulus will precede the test stimulus at ISIs of 2, 3, and 5 ms. For LICI, two suprathreshold pulses at 120% RMT will be delivered at ISIs of 50, 100, and 150 ms [46,47].

### 4.6. Participant Timeline

Following eligibility screening and informed consent, baseline assessments will be completed before randomisation. Baseline cognitive assessments will be completed during Week 0, and baseline EEG and TMS will be conducted on Day 1. Participants will then be randomised 1:1 into one of two groups, with the first active or sham iTBS session delivered immediately following randomisation. The intervention begins in Week 1, with active or sham iTBS delivered over 5 consecutive weekdays (Days 1–5). EEG and TMS will be repeated on Day 5 to evaluate short-term effects.

**Figure 2:**
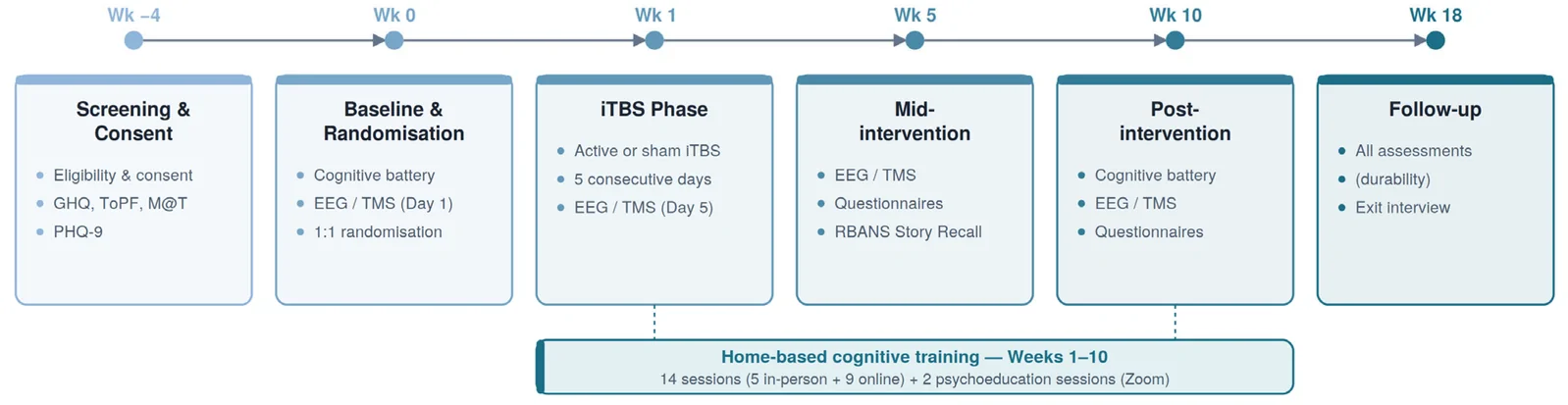
Participant timeline for the iCog.X Boost Trial, illustrating screening and consent, randomisation, intervention, and assessment timepoints across 18 weeks.

Participants will then complete 9 weeks of home-based CT (Weeks 2–10), with a mid-intervention EEG/TMS at Week 5. At the end of Week 10, post-intervention assessments will include cognitive testing, EEG, and TMS. At Week 18, all assessments will be repeated to evaluate durability of effects. The overall participant flow is summarised in Figure 2 and the Schedule of Activities in Table 4. A complete visual overview of the study design and schedule is provided in Figure S1 (Supplementary Materials).

### 4.7. Sample Size

As this is a feasibility pilot trial, the sample size was not determined by formal power calculations for hypothesis testing. Sample size determination in pilot studies is typically based on practical considerations, study logistics, and precedent rather than statistical power, with 12 participants per group recommended as a reasonable minimum [48]. Feasibility and pilot trials using iTBS and rTMS in MCI have employed sample sizes ranging from 18 to 38 participants [49]. A sample of 44 participants (22 per group) is consistent with and exceeds these precedents, and is sufficient to characterise feasibility outcomes, estimate variability in cognitive measures, generate preliminary effect size estimates, and is expected to provide preliminary neurophysiological data to inform the design of a future definitive trial. An anticipated dropout rate of ~20% has been accounted for [50], and effect size estimates will build on a pooled effect size of g = 0.36 for CT in MCI [11] to inform a future definitive trial.

### 4.8. Recruitment

**Figure 3:**
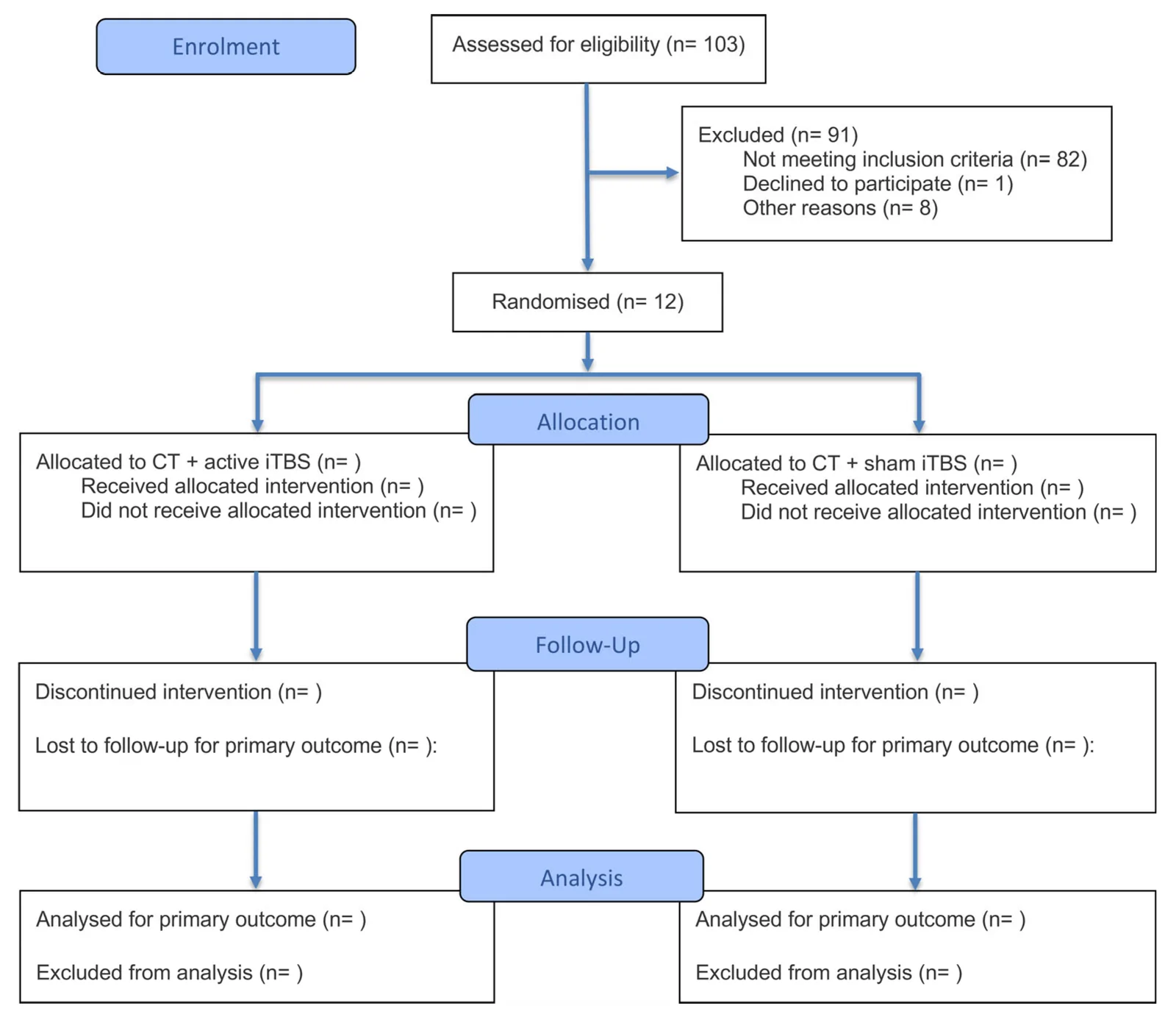
CONSORT 2025 flow diagram of participant screening, enrolment, randomised allocation to the active and sham iTBS arms, and planned follow-up and analysis. Flow diagram beyond randomised count is intentionally left blank as the trial is currently ongoing.

Participants will be recruited via the Behind the Brainwaves database (curated by GZS [50]), clinical networks (MIH, KN), social media (e.g., Facebook, Instagram, LinkedIn), community groups (Dementia Australia, StepUp for Dementia), and academic research networks. Interested individuals will complete a pre-screening questionnaire. Those meeting initial criteria will conduct a phone interview, with verbal consent recorded in REDCap. Eligible individuals will then attend an in-person screening where written informed consent is obtained. Screening includes the GHQ-12, MINI, ToPF, medical history review, and confirmation of English proficiency and MCI status using the M@T [51]. Participant flow through screening, enrolment, allocation, and follow-up is summarised in Figure 3.

### 4.9. Randomisation and Blinding

Randomisation will be implemented using REDCap with a 1:1 allocation ratio. Stratified block randomisation (block of 4 and 6) will be used, stratified by sex due to known sex differences in cognitive ageing and TMS responsiveness. The allocation sequence is computer-generated by an independent staff member and uploaded to REDCap, concealed until post-baseline assessments. Participants, outcome assessors, and data analysts will be blinded. Only the laboratory technician responsible for stimulation setup will access the allocation, solely to deliver the assigned active or sham stimulation, and will not be involved in outcome assessment or data analysis. Participants will be asked not to share their experience with others, to maintain blinding.

### 4.10. Safety

Adverse events will be monitored from first intervention to final follow-up. Serious AEs (SAEs) will be classified per NHMRC and ICH guidelines. Common iTBS side effects (e.g., headache, scalp discomfort) will be monitored. Though seizures are rare (~0.02%) [25], procedures are in place, including cessation of stimulation, and emergency medical response if required. SAEs will be reported to the Western Sydney University HREC within 24 h, with REDCap alerting the study physician automatically. Participants will be followed until AEs/SAEs resolve or stabilise. Unblinding may occur if necessary. AEs/SAEs will be summarised in annual safety reports.

### 4.11. Data Management and Monitoring

Study data will be collected using REDCap in real time. Participants will be assigned unique IDs, with identifiable information stored separately with password protection and encryption. Access will be role-restricted with audit trails. Data will be stored securely on Western Sydney University’s network and retained for 15 years post publication. Upon completion of the trial, the de-identified dataset generated during the study may be archived and will be made available from the corresponding author upon reasonable request, subject to ethical and privacy requirements. The research team will permit audits and regulatory review as required.

### 4.12. Statistical Analysis Plan

Consistent with the feasibility objectives of this trial, the primary analysis will focus on descriptive statistics, with feasibility outcomes compared against the predefined thresholds in Table 3 to guide progression to a definitive trial.

Secondary cognitive, functional, and psychological outcomes will be analysed using linear mixed-effects models (LMMs) to account for repeated measures and group differences. Fixed effects will include group (CT + iTBS, CT + sham-iTBS), time (baseline, post-iTBS, mid-intervention, post-intervention, follow-up), and their interaction. Participant ID will be modelled as a random effect. Model assumptions (e.g., normality of residuals, homogeneity of variance) will be tested and corrected as needed. Effect sizes will be estimated using Cohen’s d or f or partial η^2^ with 95% confidence intervals to inform sample size calculations for a future adequately powered trial. Analyses will be conducted using SPSS v25.0 (IBM Corp., IBM Corp., Armonk, NY, USA), Stata (StataCorp, College Station, TX, USA), or R (R Foundation for Statistical Computing, Vienna, Austria). *p*-values will be reported as nominal exploratory statistics and will not be interpreted as confirmatory evidence of efficacy. Covariates (age, sex, education) may be included in sensitivity analyses. Neurophysiological–cognitive correlations will be reported descriptively. Analyses will follow an intention-to-treat principle, with missing data handled using multiple imputation where appropriate.

### 4.13. Patient and Public Involvement

Two consumers were involved in reviewing the research materials for the CT intervention. Participants will also provide feedback on intervention acceptability and satisfaction through exit interviews, which will inform refinement of the study for a future trial.

## 5. Discussion

This protocol describes a randomised, double-blind, sham-controlled feasibility pilot trial evaluating the combined delivery of iTBS and structured cognitive training in people with MCI. The study is designed to address the gap between preliminary evidence suggesting potential cognitive benefits from CT and neuromodulation and the practical requirements for implementing a combined intervention in a future definitive trial. By prioritising feasibility, acceptability, adherence, and safety, the study will assess whether the intervention can be delivered as planned in the target population and whether predefined progression criteria are met.

A key strength is the integration of a brief iTBS protocol with a structured CT program that transitions from supervised in-person sessions to home-based training, balancing standardisation and participant support with accessibility and real-world feasibility. The inclusion of EEG and TMS measures provides an opportunity to explore neurophysiological changes that may accompany cognitive change, providing insights into biological drivers of treatment responsivity, although these analyses are exploratory and not intended to establish definitive mechanisms.

Several limitations should be considered. First, the feasibility sample is not powered to detect definitive between-group treatment effects; cognitive and neurophysiological findings will therefore be interpreted as preliminary estimates to inform the design and sample size of a future adequately powered trial. Second, eligibility criteria required for participant safety, intervention delivery, and study standardisation may limit generalisability to the broader MCI population; requirements relating to age, English proficiency, handedness, digital access, and TMS eligibility may yield a sample that is healthier or more technologically able than the wider MCI population. Third, the mixed in-person and home-based delivery model may introduce variability in the context in which CT is completed, although online completion will be automatically recorded and adherence patterns monitored. Finally, because the intervention combines iTBS with a structured cognitive training program, psychoeducation, and remote support, any subsequent signals of cognitive change cannot be attributed to a single component.

Despite these limitations, the study is designed to provide practical evidence regarding recruitment, retention, adherence, acceptability, and safety, together with preliminary estimates of cognitive and neurophysiological outcomes, which will refine the intervention, outcome selection, delivery procedures, and sample-size assumptions for a future definitive randomised controlled trial.

## 6. Ethics and Dissemination

Ethical approval has been granted by the Western Sydney University HREC (H16500), and the study will be conducted in accordance with the National Statement on Ethical Conduct in Human Research and Good Clinical Practice guidelines. All participants will provide written informed consent before enrolment. Confidentiality will be ensured through secure data storage and de-identification in REDCap. Participants may withdraw at any time without consequence.

Findings from the trial will be disseminated through peer-reviewed publications, conference presentations and academic theses. A lay summary of results will be provided to participants following study completion.

## 7. Trial Registration

This trial was registered on 29 August 2025 with the Australia and New Zealand Clinical Trials Registry (ACTRN12625000952448): https://anzctr.org.au/Trial/Registration/TrialReview.aspx?id=390009 (accessed on 17 August 2026).

## 8. Trial Status

This trial is currently in the recruitment phase.

## Figures and Tables

**Table 1:** Predefined feasibility indicators and stop/go decision criteria.

Indicator	Proceed	Proceed with Modifications	Do Not Proceed
**Recruitment-related variables**			
Recruitment rate	At least 2 participants per month can be recruited	At least 1 participant per month can be recruited	Less than 1 participant per month can be recruited
Eligibility yield	One or more in four screened participants meets eligibility criteria (≥25%)	At least one in six screened participants meets eligibility criteria	Less than one in six screened participants meets eligibility criteria
**Intervention-related variables**			
Adherence	≥80% of participants complete ≥ 80% of prescribed iTBS and CT sessions	≥65% of participants complete ≥ 80% of prescribed iTBS and CT sessions	<65% of participants complete ≥ 80% of prescribed iTBS and CT sessions
Tolerability	≥75% of withdrawals are not attributable to intervention-related adverse events	60–74% of withdrawals are not attributable to intervention-related adverse events	<60% of withdrawals are not attributable to intervention-related adverse events
Acceptability	≥70% of participants rate the intervention as acceptable or highly acceptable on post-intervention scale	50–69% of participants rate the intervention as acceptable	<50% of participants rate the intervention as acceptable
**Attrition**			
Retention	<20% participant attrition at final follow-up (Week 18)	20–30% attrition at final follow-up	>30% attrition at final follow-up

**Table 2:** Inclusion and exclusion criteria.

Inclusion Criteria	Exclusion Criteria
– Aged 55–85 years – MCI based on Winblad criteria [30] – Subjective cognitive complaints via a semi-structured clinical interview – Objective cognitive decline (M@T score 27–37) OR scoring at least 1.0–1.5 SDs below age-adjusted population norms and/or estimated premorbid ability based on the ToPF – Preserved or minimal iADL impairment based on the HBA-FAQ and study neuropsychologist discretion. – Right-handed (Edinburgh Handedness Inventory) – Fluent in English (reading, writing, speaking) – Normal or corrected-to-normal vision and hearing – Access to a personal desktop or laptop with full internet access	– Prior exposure to TMS within the past 12 months – M@T score < 27 – Study neuropsychologist discretion regarding cognitive and/or functional status based on neuropsychometric performance. – Current symptoms of major depression, defined as a PHQ-9 score ≥ 10 and meeting criteria for a current depressive episode on the MINI interview. – Contraindications to TMS [29]. – Alcohol consumption within 24 h of assessments – Current smokers – Low digital literacy as determined by difficulty navigating basic computer tasks during initial screening. – Diagnosed psychiatric affective and non-affective disorders including dissociative disorder, obsessive compulsive disorder, personality disorders, schizophrenia, bipolar disorder – History of significant head trauma resulting in loss of consciousness > 30 min, retrograde amnesia, or post-traumatic concussion syndrome, or any injury with persistent neurological sequelae or known structural brain abnormalities

**Note:** HBA-FAQ = Healthy Brain Ageing Functional Assessment Questionnaire; M@T = Memory Alteration Test; PHQ-9 = Patient Health Questionnaire-9; ToPF = Test of Premorbid Functioning.

**Table 3:** Overview of the iCog.X Boost cognitive training program: session structure, cognitive focus, strategies, and home exercises.

Session	Cognitive Focus	Training Activities	Strategies Used	Weekly Home Exercise
1	Introduction to Cognitive Training	Platform familiarisation, delayed matching, auditory memory recall	Chunking, retrieval practice, visual imagery	Introduce cognitive training principles, establish baseline, and teach foundational memory strategies
2	Attention & Processing Speed	Visual search, dual-task (memory + attention)	Focused attention, dual-task training	Improve selective attention, processing speed, and task coordination
3	Memory (Auditory & Visual)	Word-pair recall, story recall, visual search	Association elaboration, Method of Loci	Strengthen encoding and retrieval processes, enhance associative memory
4	Reasoning (Inductive & Deductive)	Pattern recognition, rule identification	Logical deduction, analogy-based reasoning	Develop reasoning strategies and cognitive flexibility
5	Problem-Solving & Decision-Making	Real-life problem-solving tasks, strategic planning exercises	Stepwise problem-solving, decision trees	Enhance problem-solving skills and apply strategies to real-world situations
6	Attention (Divided & Selective)	N-back task, divided attention in ADLs	Prioritization, response inhibition	Strengthen divided attention and working memory
7	Complex Attention & Executive Functioning	Dual-task (memory + attention)	Task-switching, cognitive flexibility	Reinforce attention strategies and adaptive executive functioning
8	Visual Memory	Visual memory in ADLs	Mnemonic reinforcement, environmental cues	Generalise memory strategies to everyday activities
9	Auditory Memory	Auditory recall tasks in ADLs	Active listening, verbal rehearsal	Improve auditory memory
10	Dual-Task Training	Memory + attention, ADL-based attention	Divided attention practice, strategy switching	Reinforce multi-tasking and integration of memory and attention skills
11	Advanced Reasoning & Problem-Solving	Inductive reasoning, problem-solving tasks	Hypothesis testing, inferential thinking	Apply problem-solving techniques to novel contexts
12	Decision-Making & Planning	Deductive reasoning, complex decision-making in ADLs	Mental simulation, pre-decision frameworks	Strengthen logical reasoning and structured decision-making
13	Integrated Problem-Solving	Dual-task (reasoning + memory), problem-solving in ADLs	Multi-step problem-solving, adaptive reasoning	Apply advanced problem-solving strategies across domains
14	Strategy Consolidation	Recap of all cognitive strategies, transfer to daily activities	Meta-cognitive reflection, self-monitoring	Ensure generalisation and long-term retention of trained skills

**Table 4:** Schedule of activities for screening, baseline, endpoint, and follow-up timepoints.

Assessment Schedule		Screening (−4 wks)	Baseline (0 wks)	Post-iTBS (1 wk)	Midpoint (5 wks)	Endpoint (10 wks)	Follow-Up (18 wks)
**Screening**	Inclusion/Exclusion Criteria	X					
	Informed Consent	X					
	GHQ	X					
	Premorbid Function (ToPF)	X					
	M@T	X				X	X
	CVLT-II Short Form	X					
	HBA-FAQ	X	X			X	X
	MINI	X					
**Treatment Period: Intervention (Weeks 1–10)**							
**Feasibility**	Exit Interview *						X
**Neuropsychological Test Battery**	CVLT-II Standard Form		X			X	X
	WAIS-IV Similarities		X			X	X
	WAIS-IV Digit Span		X			X	X
	WAIS-IV Coding		X			X	X
	D-KEFS TMT		X			X	X
	Corsi Block-Tapping Test		X			X	X
	RBANS Story Recall		X		X	X	X
	EPT		X			X	X
**Neurophysiological Measures**	TMS Measurements		X	X	X	X	X
	EEG Measurements		X	X	X	X	X
**Lifestyle**	PSQI		X		X	X	X
**Functioning**	EQ-5D-5L		X		X	X	X
**Psychological Health**	PHQ-9	X	X		X	X	X
	GAD-7		X		X	X	X

**Abbreviations:** Corsi Block-Tapping Test = visuospatial working memory task; CVLT-II = California Verbal Learning Test—Second Edition; D-KEFS TMT = Delis–Kaplan Executive Function System Trail Making Test; EEG = Electroencephalography; EPT = Everyday Problems Test; EQ-5D-5L = EuroQol 5 Dimensions 5-Level; GAD-7 = Generalised Anxiety Disorder—7 Items; GHQ = General Health Questionnaire; HBA-FAQ = Healthy Brain Ageing Functional Assessment Questionnaire; M@T = Memory Alteration Test; MINI = Mini International Neuropsychiatric Interview; PHQ-9 = Patient Health Questionnaire—9 Items; PSQI = Pittsburgh Sleep Quality Index; RBANS = Repeatable Battery for the Assessment of Neuropsychological Status (Story Recall); TMS = Transcranial Magnetic Stimulation; ToPF = Test of Premorbid Function; WAIS-IV = Wechsler Adult Intelligence Scale-Fourth Edition (Similarities, Digit Span, Coding). Note. * Exit interview to be conducted at follow-up or whenever the participant exits the trial if withdrawing prior to completion. X indicates the assessment or procedure is performed at that timepoint.

## Data Availability

Upon completion of the trial, the de-identified dataset generated during the study may be archived and will be made available from the corresponding author upon reasonable request, subject to ethical and privacy requirements.

## Supplementary Materials

The following supporting information can be downloaded at: https://www.mdpi.com/article/10.3390/mps9050130/s1, Table S1: Cognitive, functional, and psychological outcome measures: rationale and psychometric properties [52,53,54,55,56,57,58,59,60,61,62,63,64,65,66]. Figure S1: Complete overview of the study design, timeline, intervention phases, and assessment schedule of the iCog.X Boost Trial.

## Abbreviations

The following abbreviations are used in this manuscript:

AD	Alzheimer’s disease
ADL	Activities of daily living
AE	Adverse event
CT	Cognitive training
CVLT-II	California Verbal Learning Test—Second Edition
D-KEFS TMT	Delis–Kaplan Executive Function System Trail Making Test
DLPFC	Dorsolateral prefrontal cortex
EEG	Electroencephalography
EMG	Electromyography
EOG	Electrooculogram
EPT	Everyday Problems Test
EQ-5D-5L	EuroQol 5 Dimensions 5-Level
ERP	Event-related potential
FDI	First dorsal interosseous
GAD-7	Generalised Anxiety Disorder—7 Items
GHQ	General Health Questionnaire
HBA-FAQ	Healthy Brain Ageing Functional Assessment Questionnaire
HREC	Human Research Ethics Committee
iADL	Instrumental activities of daily living
ICH	International Council for Harmonisation
iTBS	Intermittent theta burst stimulation
LICI	Long-interval intracortical inhibition
LMM	Linear mixed-effects model
M@T	Memory Alteration Test
MCI	Mild cognitive impairment
MEP	Motor-evoked potential
MINI	Mini International Neuropsychiatric Interview
NHMRC	National Health and Medical Research Council
PHQ-9	Patient Health Questionnaire—9 Items
PSQI	Pittsburgh Sleep Quality Index
RAAA	Revised Aligned Artefact Average
RBANS	Repeatable Battery for the Assessment of Neuropsychological Status
RCT	Randomised controlled trial
REDCap	Research Electronic Data Capture
RMT	Resting motor threshold
rTMS	Repetitive transcranial magnetic stimulation
SAE	Serious adverse event
SICI	Short-interval intracortical inhibition
TMS	Transcranial magnetic stimulation
ToPF	Test of Premorbid Functioning
WAIS-IV	Wechsler Adult Intelligence Scale—Fourth Edition
