# Supplementary material for: Intermittent Theta Burst Stimulation Combined with Cognitive Training in Mild Cognitive Impairment: Protocol for an 18-Week Randomised, Double-Blind, Sham-Controlled Feasibility Pilot Study (The iCog.X Boost Trial)
Source: Methods Protoc. 2026 Sep 1;9(5):130. doi: 10.3390/mps9050130 (PMC13600087; doi:10.3390/mps9050130)
Supplement: Supplementary file 1 [file mps-09-00130-s001.zip › Supplementary Table S1.pdf]

Supplementary Table S1. Cognitive, functional, and psychological outcome measures: rationale and psychometric properties

Outcome Measure	Cognitive Domain	Psychometric Properties	References
**California Verbal Learning Test-II (CVLT-II)**	Verbal memory	Test-retest reliability = 0.80-0.84; Cohen's d for practice effects = -0.01 to 0.18; Sensitivity = 90.2%; Specificity = 84.2% for detecting MCI	(1, 2)
**RBANS Story Recall Subtest**	Episodic memory	AUC = 0.90 (Delayed Memory Index), AUC = 0.88 (Total Score); validated for early Alzheimer's disease	(3)
**Corsi Block-Tapping Test**	Visuospatial working memory	Medium to large effect sizes; good discriminative validity for MCI	(4)
**WAIS-IV Digit Span**	Auditory attention and working memory	Differentiates MCI subtypes and healthy controls	(5)
**TMT - DKEFS (Conditions 2 and 5)**	Processing speed and executive function	Sensitivity = 49.6%; Specificity = 76.2% for predicting MCI to AD conversion	(6)
**WAIS-IV Coding Subtest**	Processing speed	Sensitivity = 88%; Specificity = 76%; associated with iADL performance	(7)
**Everyday Problems Test (EPT)**	Everyday problem-solving / functional reasoning	Validated for detecting functional reasoning deficits in older adults	(8)
**WAIS-IV Similarities Subtest**	Verbal abstract reasoning	Internal consistency = 0.84-0.94; Test-retest reliability = 0.87-0.96	(9)
**HBA Functional Assessment**	Instrumental activities of daily living (iADLs)	Strong predictive validity for functional decline	(10)

**Questionnaire (HBA-FAQ)**			
**PHQ-9**	Depressive symptoms	Internal consistency = high; Sensitivity = 89%; Specificity = 71% for MCI	(11)
**GAD-7**	Anxiety symptoms	Validated in older adults and MCI populations	(12)
**Pittsburgh Sleep Quality Index (PSQI)**	Sleep quality	Widely used in middle-aged Australian cohorts	(13, 14)
**EQ-5D-5L**	Health-related quality of life	Brief, validated; includes 5 domains and 0-100 self-rated VAS	(15)

References:

1. Woods SP, Delis DC, Scott JC, Kramer JH, Holdnack JA. The California Verbal Learning Test–second edition: Test-retest reliability, practice effects, and reliable change indices for the standard and alternate forms. Archives of clinical neuropsychology. 2006;21(5):413-20.
2. Rabin LA, Paré N, Saykin AJ, Brown MJ, Wishart HA, Flashman LA, et al. Differential memory test sensitivity for diagnosing amnestic mild cognitive impairment and predicting conversion to Alzheimer's disease. Aging, Neuropsychology, and Cognition. 2009;16(3):357-76.
3. Karantzoulis S, Novitski J, Gold M, Randolph C. The Repeatable Battery for the Assessment of Neuropsychological Status (RBANS): Utility in detection and characterization of mild cognitive impairment due to Alzheimer's disease. Archives of clinical neuropsychology. 2013;28(8):837-44.
4. Liebermann-Jordanidis H, Roheger M, Boosfeld L, Franklin J, Kalbe E. Which test is the best to assess visuo-cognitive impairment in patients with Parkinson's disease with mild cognitive impairment and dementia? A systematic review and meta-analysis. Journal of Parkinson's Disease. 2022;12(6):1749-82.
5. Emrani S, Libon DJ, Lamar M, Price CC, Jefferson AL, Gifford KA, et al. Assessing working memory in mild cognitive impairment with serial order recall. Journal of Alzheimer's disease. 2018;61(3):917-28.
6. Ashendorf L, Jefferson AL, O'Connor MK, Chaisson C, Green RC, Stern RA. Trail Making Test errors in normal aging, mild cognitive impairment, and dementia. Archives of Clinical Neuropsychology. 2008;23(2):129-37.
7. Galvin JE, Tolea MI, Moore C, Chrisphonte S. The Number Symbol Coding Task: A brief measure of executive function to detect dementia and cognitive impairment. PLoS One. 2020;15(11):e0242233.
8. Willis SL, Allen-Burge R, Dolan MM, Bertrand RM, Yesavage J, Taylor JL. Everyday problem solving among individuals with Alzheimer's disease. The Gerontologist. 1998;38(5):569-77.
9. Szczepkowski KV, Demakis GJ. Wechsler Adult Intelligence Scale. In: Zeigler-Hill V, Shackelford TK, editors. Encyclopedia of Personality and Individual Differences. Cham: Springer International Publishing; 2020. p. 5746-50.

10. Lin P, LaMonica HM, Naismith SL, Mowszowski L. Identifying subtle functional change in individuals with mild cognitive impairment: development and validation of the Healthy Brain Ageing–Functional Assessment Questionnaire. Aging, Neuropsychology, and Cognition. 2023;30(4):536-54.

11. Kroenke K, Spitzer RL, Williams JB. The PHQ-9: validity of a brief depression severity measure. Journal of general internal medicine. 2001;16(9):606-13.

12. Williams N. The GAD-7 questionnaire. Occupational medicine. 2014;64(3):224-.

13. Buysse DJ, Reynolds CF, Monk TH, Berman SR, Kupfer DJ. The Pittsburgh Sleep Quality Index: a new instrument for psychiatric practice and research. Psychiatry Res. 1989;28:193-213.

14. Turner JA, Laslett LL, Padgett C, Lim CK, Taylor B, van der Mei I, et al. Disease-modifying therapies do not affect sleep quality or daytime sleepiness in a large Australian MS cohort. Multiple Sclerosis and Related Disorders. 2023;78:104902.

15. van Hout B, Mulhern B, Feng Y, Shah K, Devlin N. The EQ-5D-5L value set for England: response to the “quality assurance”. Value in Health. 2020;23(5):649-55.
